# Correlation of the expression of vascular endothelial growth factor and its receptors with microvessel density in ovarian cancer

**DOI:** 10.3892/ol.2013.1349

**Published:** 2013-05-15

**Authors:** LIMEI WANG, XIAOYAN LIU, HONG WANG, SHUHE WANG

**Affiliations:** 1 Departments of Obstetrics and Gynecology, Beijing General Army Hospital, Beijing 100700, P.R. China; 2 Medical Record Management, Beijing General Army Hospital, Beijing 100700, P.R. China; 3 Ultrasound, Beijing General Army Hospital, Beijing 100700, P.R. China

**Keywords:** vascular endothelial growth factor, Flt-1 receptor, KDR receptor, oophoroma, ascites

## Abstract

The present study aimed to investigate the correlation between the expression of vascular endothelial growth factor (VEGF) and its receptors, the Flt-1 and KDR proteins, with clinical pathology and microvessel density (MVD) in ovarian cancer tissue. The protein expression levels of VEGF and its receptors, Flt-1 and KDR/Flk-1, were detected in 48 cases of ovarian cancer using the streptavidin-biotin complex (SABC) immunohistochemical method, and tumor MVD was evaluated using F8 factor (FVIII-RA). The expression of the VEGF, Flt-1 and KDR proteins was not significantly associated with the pathological type, extent of differentiation or clinical stage of ovarian cancer. However, the co-expression of VEGF and Flt-1 was markedly correlated with differentiation and clinical stage (P<0.01). The co-expression levels of VEGF and receptor Flt-1 in malignant neoplasms with lymph node metastasis was significantly higher compared with malignant neoplasms without lymph node metastasis (P<0.05). The expression level of KDR in patients with hepatic metastasis was significantly higher compared with patients without hepatic metastasis (P<0.05). The co-expression level of VEGF and KDR in patients with hepatic metastasis was significantly higher compared with patients without hepatic metastasis (P<0.05) and the Flt-1 expression level in patients with ascites <1,000 ml was significantly lower than that in patients with ascites ≥1000 ml (P<0.05). The mean MVD of VEGF- and KDR-positive patients was significantly higher compared with VEGF- and KDR-negative patients (P<0.05). The expression of VEGF and its receptors is involved in the malignant transformation of ovarian tumors, tumor progression and metastasis, as well as ascites formation and angiogenesis.

## Introduction

The growth, development, invasion and metastasis of solid tumors are dependent on angiogenesis. Vascular endothelial growth factor (VEGF) is the main angiogenesis factor and is able to increase vasopermeability and promote neovascularization. VEGF provides essential nutrients for tumor growth and network matrixes for tumor cell invasion and metastasis, and has important roles in ascites generation and malignant tumor progression. VEGF has important physiological roles created mainly when combining with specific receptors (1–3).

Ovarian cancer is a type of euangiotic tumor, which has numerous pathological types and no rational clinical symptoms. It is difficult to identify early, but may exhibit extensive peritoneal metastasis. Ovarian cancer is highly malignant and has a poor prognosis. The mortality rate of ovarian cancer is greater than the total mortality rate of cervical carcinoma and endometrial carcinoma. It is a predominant gynecological tumor. Evidence suggests that the abnormal expression of VEGF in ovarian cancer is closely associated with tumor invasion and metastasis (4–6). In ovarian tumors with rapid growth, there are vascular distributions, while slow-growing tumor vessels are only concentrated in peripheral vessels. Animal experiments have demonstrated that fine avascular tumors with ovarian cancer metastasizing into the peritoneum do not grow. However, following neovascularization, the tumors grow rapidly. VEGF may promote not only angiopoiesis, but also tumor proliferation by the paracrine or autocrine mechanisms. Tumor microvessel density (MVD) is a valuable prognostic factor for advanced ovarian cancer. Hefler *et al* (5) considered that serum VEGF levels were closely associated with the prognosis of ovarian cancer. Although there are studies on the correlation of VEGF with ovarian cancer, the results of studies concerned with the correlation of the expression of VEGF and its receptors with the clinical pathology of ovarian cancer remain inconsistent. In addition, the pathogenesis remains unclear, and studies investigating the resistance to ovarian cancer angiopoiesis through the use of VEGF have only just commenced. Therefore, the aim of the present study was to investigate the correlation of the expression of VEGF and its receptors with clinical pathology and MVD in ovarian cancer tissues by conducting a case-control study, and to further investigate the role of VEGF in ovarian cancer invasion and metastasis, as well as in ascites and angiogenesis.

## Subjects and methods

### Subjects

A total of 48 patients with ovarian cancer were selected from hospitalized patients receiving surgery at the General Hospital of Beijing Military Area Command, Beijing, China, between January 2000 and June 2004. The ages of the patients ranged between 14 and 70 years old, and the mean age was 48.4 years old. Among the patients, there were 41 cases of epithelial cancer (including 24 cases of serous carcinoma, seven cases of mucinous carcinoma and 10 cases of adenocarcinoma anaplastic) and seven cases of non-epithelial tumors (including five cases of granulose cell tumors, one case of dysgerminoma and one case of an endodermal sinus tumor). Moreover, 14 cases were highly and moderately differentiated and 24 cases were poorly differentiated. Prior to surgery, an abdominal CT was performed to identify hepatic metastasis, and was confirmed by intra-operative exploration. Standardized cytoreductive surgery was performed. The clinical staging complied with the staging criteria prepared by the International Federation of Gynecology and Obstetrics (FIGO) in 1985 (7). Among all the cases, there were 16 cases in stages I and II and 32 cases in stages III and IV. All tissue specimens were obtained during surgery, and the primary lesions of the tumors were collected and fixed with 10% formalin for routine histological and immunohistochemical examination. The present study was conducted in accordance with the declaration of Helsinki and with approval from the Ethics Committee of Beijing General Army Hospital. Written informed consent was obtained from all participants.

### Immunohistochemical examination

The VEGF antibody was a gift from Professor Yang Zhihua of the Institute of Oncology, Chinese Academy of Medical Sciences (Beijing, China). The antibody immunohistochemical streptavidin-biotin complex (SABC) kit and rabbit anti-human Flt-1 and anti-KDR antibodies were purchased from Wuhan Boster Company (Wuhan, China). The tissues were conventionally fixed with 10% formalin, then dehydrated with alcohol, embedded with paraffin wax and continuously sectioned into slices 4-μm thick. One slice was used for the pathological examination and the other two slices were used to detect VEGF and receptor protein expression, respectively. In each experiment, the negative control group was identical, with phosphate-buffered saline (PBS) used to replace the first antibody for staining. For the positive control group, the provided positive slice was used for staining. Brown granules in the cytoplasm of the tumor and vascular endothelial cells were treated as positive and the expression intensity was relative to the standard (7). The observed results were judged by two blinded pathology experts.

### Statistical analysis

Data were processed using the SPSS 10.0 software package (SPSS Inc., Chicago, IL, USA). The Chi-squared test, exact probability for four-fold tables and non-parametric rank sum test (Kruskal-Wallis) were used to compare expression levels. P<0.05 was considered to indicate a statistically significant difference.

## Results

### Expression of VEGF, Flt-1 and KDR/Flk-1

**Figure 1.**
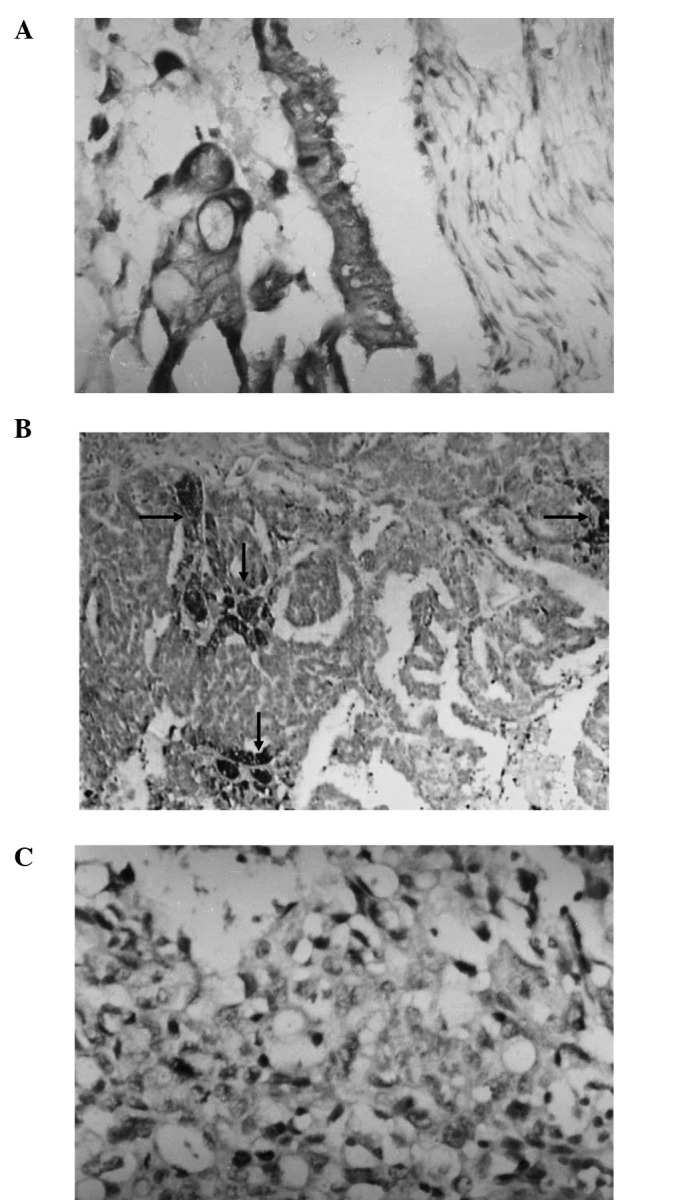
(A) Results of immunohistochemical staining for VEGF in ovarian cancer tissues. Microscopy showed that the tumor cell cytoplasm was stained, brown granules were visible and that consequently, VEGF expression was markedly positive. (B) Results of immunohistochemical staining for Flt-1 in ovarian cancer tissues. The tumor cells and stroma vessels were stained and the degree of vascular positive staining (arrows) was stronger. (C) Results of immunohistochemical staining for KDR in ovarian cancer tissues. Microscopy showed that brown granules were visible in the tumor cell cytoplasm, the nucleus was blue and that all the tumor cells were stained. VEGF, vascular endothelial growth factor. (A–C) Magnification, ×200.

VEGF, Flt-1 and KDR expression was present in the cytoplasm and vessels of the ovarian cancer tissues, exhibited as focal or diffuse expression. The expression level of VEGF in ovarian cancer was 66.7% (32/48). In total, 54.2% (26/48) of the staining was markedly positive (>++), while 12.5% (6/48) was weakly positive (+). The expression level of Flt-1 was 58.3% (28/48), and the expression level of KDR was 43.8% (21/48). The co-expression level of VEGF and Flt-1 was 92.9% (26/28), and the co-expression level of VEGF and KDR was 90.5% (19/2; Fig. 1).

### Correlation of VEGF, Flt-1 and KDR expression with clinical pathology and stage

The co-expression levels of VEGF and Flt-1 protein in the epithelial and non-epithelial tumors were 51.2 and 71.4%, respectively, and the difference in the pairwise comparison was not significant (P= 0.58). The co-expression levels of VEGF and Flt-1 in the highly and moderately differentiated and the poorly differentiated tumors were 28.6 and 64.7%, respectively, and the difference was significant (P=0.02). In addition, the co-expression levels of VEGF and Flt-1 in the tumors of FIGO stages I and II and in stages III and IV were 25.0 and 68.7%, respectively, and the difference was significant (P= 0.005). The co-expression level of VEGF/KDR was not associated with the tumor pathological type, extent of differentiation or clinical stage (Table I).

### Correlation of VEGF, Flt-1 and KDR expression with clinical metastasis and ascites

The expression levels of VEGF and Flt-1 in the patients with lymph node metastasis were 85.7 and 78.6%, respectively, evidently higher than those in the patients without lymph node metastasis; the two differences were significant (P=0.009; P=0.02). The Flt-1 expression level of the 18 cases with ascites <1000 ml was 45.2%, evidently lower than that of the patients with ascites ≥1000 ml (78.3%); the difference was significant (P=0.02). For the KDR expression level, there was a significant difference between the patients with hepatic metastasis and the patients without hepatic metastasis (P= 0.02), while the co-expression level of VEGF and KDR in the patients with hepatic metastasis was significantly increased (P=0.005; Table II).

### Correlation of VEGF and receptor expression with MVD

**Figure 2.**
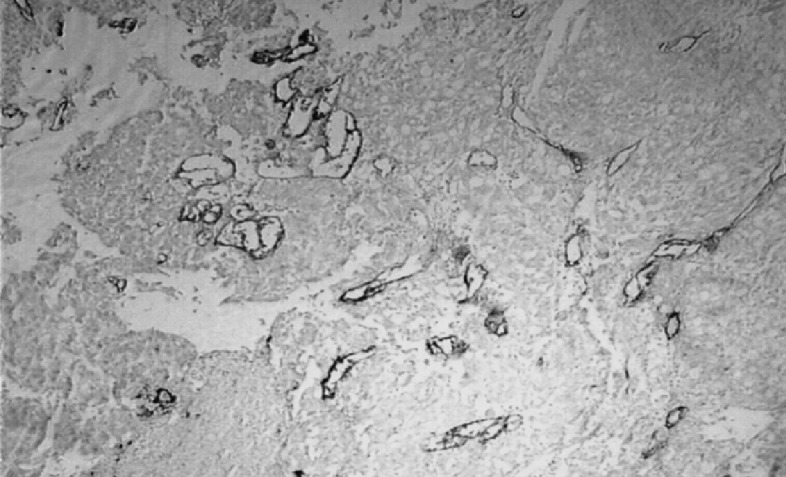
MVD of patients with positive VEGF/KDR expression (F8 was used as a vascular endothelial cell marker, SABC staining). Microscopy showed that the microvessels were abundant, and blood capillary clusters and slit-shaped vessels were visible (magnification, ×100). MVD, microvessel density; VEGF, vascular endothelial growth factor; SABC, streptavidin-biotin complex.

Among the 32 VEGF-positive patients, the mean MVD was 19.51±8.69. Compared with the mean MVD of the 16 VEGF-negative patients (12.68±4.04), the difference was significant (P=0.01). The mean MVD of the Flt-1-positive patients was 19.19±9.53. Compared with mean MVD of the Flt-1-negative patients (17.16±6.74), the difference was not significant (P=0.54). The mean MVD of the KDR-positive patients was 21.64±8.63, and that of the KDR-negative patients was 17.06±7.87. There was a significant difference between these two groups (P=0.03; Table III; Fig. 2).

## Discussion

VEGF, also known as vascular permeability factor, is a multi-functional factor, with a highly conservative protein structure. When its glycoprotein monomers are combined by disulfide bonds to form a dimer, it becomes biologically active. The Flt-1 and KDR proteins are VEGF-specific receptors, belonging to the type III tyrosine kinase receptors. VEGF has important physiological roles created mainly when combining with specific receptors (3). A number of studies have demonstrated that VEGF is the main positive regulator in the process of tumor angiogenesis, and that it is involved in the occurrence and development of tumors by promoting angiogenesis, as well as being associated with the degree of malignancy of tumors (8–10). The growth of solid tumors is divided into the non-vascular pre-invasion stage and the vascularization invasion growth stage. In the vascularization stage, tumors begin to grow rapidly. For the growth of the germinal center and tumors, oxygen and nutritional supplies are required from the vessels, otherwise hypoxia and necrosis will occur. In order to maintain the unlimited invasive growth of malignant tumors, the tumors must continuously and extensively conduct angiopoiesis. Numerous study results have demonstrated that VEGF is the main positive regulator in the process of tumor angiogenesis and that it is involved in the occurrence and development of tumors by promoting angiogenesis (10). The present immunohistochemical results showed that the majority of tissue samples expressing VEGF receptor exhibit positive VEGF expression. The consistent co-expression of VEGF and the VEGF receptor indicates that in ovarian cancer, VEGF not only indirectly promotes tumor cell growth by affecting the receptor on the vascular endothelial cells to induce angiopoiesis, but also directly promotes tumor cell growth by affecting the receptor on the tumor cells. Verheul *et al* indicated that VEGF is involved in tumor occurrence and development using the paracrine or autocrine mechanisms (11). VEGF is also closely related to ovarian tumor cell proliferation. The PCR results of Shen *et al* demonstrated that VEGF was unrelated to the pathological type, but closely associated with the extent of differentiation. The VEGF expression level in poorly-differentiated cancers was 100% (markedly positive level, 83.3%), and the expression level in the highly- and moderately-differentiated cancers was 95.1% (markedly positive level, 34.8%; P=0.0004) (12). Clinical stage is associated with the degree of the malignancy of the tumors. In the present study, the expression of VEGF and its receptors was unrelated to the clinical stage of the tumor, which was not the expected result. This was possibly due to the small number of selected cases; thus it is necessary to conduct further future studies with larger sample sizes. In addition, it was observed in the present study that the co-expression level of VEGF and its receptor Flt-1 was significantly higher in poorly-differentiated tumors compared with highly- and moderately-differentiated tumors. The more advanced the tumor clinical stage was, the higher the co-expression level of Flt-1 and VEGF; this is consistent with the expected result. It appears that VEGF interacts with the Flt-1 receptor to promote malignant transformation and tumor progression.

One of the clinical characteristics of ovarian cancer is that it readily forms a large quantity of ascites. In the majority of cases, when ovarian cancer is identified, extensive metastasis in the abdominopelvic cavity has occurred, which increases the difficulty of performing surgery and thus affects the patient prognosis. The mechanisms and factors that affect ascites formation and abdominopelvic cavity metastasis in ovarian cancer remain unclear. A large number of animal experiments have suggested that, as a specific endothelial cell mitogen, VEGF is the main angiogenesis factor. VEGF is able to increase vasopermeability, promote neovascularization and is important in tumor vascular endothelial cell proliferation and migration, as well as ascites generation (13,14). Fujimoto *et al* (15) indicated that tumorous ascites formation is due to tumor-derived VEGF affecting the tumor vessels and host vessels via paracrine effects, which increase vasopermeability and cause mass extravasation of the plasma protein fluid. In the present study, the VEGF and Flt-1 receptor expression levels were increased in patients with a large quantity of ascites and positive peritoneal cytology, while KDR expression was not correlated with ascites, indicating that VEGF promotes malignant ascites generation, possibly by combining with the Flt-1 receptor.

During tumor growth, the levels of angiogenesis factors secreted by the tumor cells are markedly increased, thus significantly increasing tumor angiogenesis and speeding up tumor progression, which manifests as the features of metastasis (?). Studies suggest that avascular, resting cells in micrometastatic foci are able to remain latent in the body for a longer period of time. The proliferation rate of tumor cells in the dormancy period is the same as that of the tumor growth, and the main difference is that the death rate of the former is increased, causing angiogenesis to decrease (7). It has been proposed that VEGF expression is associated with the malignant behavior of ovarian cancer. Patients with high VEGF expression more frequently undergo lymph node and hepatic metastasis compared with VEGF-negative patients (14,16). In the present study, the VEGF expression level in the patients with lymph node metastasis was 85.7%, significantly higher than that in the patients without lymph node metastasis (40%; P= 0.0009) However, there was no significant difference between the patients with hepatic metastasis, which was possibly associated with the smaller number of cases of patients with hepatic metastasis. The Flt-1 expression level in the patients with lymph node metastasis was 71.3%, significantly higher than that in the patients without lymph node metastasis (40%; P= 0.02). VEGF may have an important role in lymph node metastasis when combined with Flt-1. In addition, the KDR expression level and the co-expression level of VEGF and KDR in patients with hepatic metastasis was significantly higher compared with patients without hepatic metastasis (P=0.02 and P=0.005, respectively), indicating that VEGF promoted the hepatic metastasis of ovarian cancer by combining with KDR and was associated with the hematogenous metastasis of ovarian cancer.

As a morphological basis of tumor growth and development, angiogenesis provides nutrition and oxygen for tumor cells to promote rapid proliferation, and microvessels are present at high-densities. In the present study, F8 was selected as a vascular endothelial marker, and the results showed that the mean MVD of patients with VEGF expression was 19.51±8.69, significantly higher compared with patients without VEGF expression (12.68±4.049; P<0.02). The corresponding MVD of the patients with VEGF expression was high. Furthermore, it was also revealed that the MVD of the patients with positive KDR expression was 21.64±8.63, significantly higher than that of the KDR-positive patients (17.06±7.87; P=0.035), while there was no significant difference in the mean MVD between the patients with and without Flt-1 expression, indicating that VEGF promotes ovarian tumor angiogenesis mainly by interacting with KDR. Shalaby *et al* (17) showed that for mice lacking the gene encoding KDR/Flk-1, embryonic endothelial cell differentiation was hindered and vessels could not be formed. Homologous mice lacking the gene encoding Flt-1 were unable to inhibit endothelial cell differentiation. Although angiopoiesis occurred, the function of the formed vessels was seriously damaged. One study (7) demonstrated that KDR is important in endothelial cell differentiation, mitosis and angiogenesis, and that it was the main angiogenesis regulator, whereas Flt-1 receptor was mainly involved in interactions between endothelial cells or between the endothelium and ECM, presenting clear vascularization and increased vasopermeability, which was consistent with the conclusions of the present study.

VEGF participates in multiple mechanisms by combining with the corresponding receptors. VEGF promotes ovarian cancer cell growth, angiopoiesis and distant metastasis. VEGF and its receptors are able to act as indicators for predicting the potential for tumor metastasis and malignant ascites. In addition, the vascular dependence of malignant tumor growth and metastasis indicates that it is feasible to inhibit these processes by inhibiting tumor angiopoiesis, thus revealing a second field of tumor treatment (18–20). Anti-angiogenesis treatments are able to not only block tumor growth, but also more markedly inhibit tumor metastasis. The present study demonstrated the significance of the role of VEGF in tumors. With VEGF and its receptors as targets, it is feasible to prepare corresponding antagonists and inhibitors to suppress VEGF synthesis and secretion, hinder the combination of VEGF with its receptors or inhibit receptor expression to block the promotion of tumor growth and metastasis.

## Figures and Tables

**Table I.** Correlation of VEGF, Flt-1 and KDR expression with clinical significance in ovarian cancer.

Category	Cases, n	VEGF		Flt-1		KDR		VEGF/Flt-1		VEGF/KDR	
		n (%)	P-value	n (%)	P-value	n (%)	P-value	n (%)	P-value	n (%)	P-value
Pathological type											
Epithelial tumor	41	27 (65.8)	0.88	24 (58.8)	0.73	17 (41.5)	-	21 (51.2)	-	16 (39.0)	-
Non-epithelial tumor	7	5 (71.4)		4 (57.1)		4 (57.1)	0.73	5 (71.4)	0.58	3 (42.8)	0.80
Tissue differentiation											
G1, G2	14	8 (57.1)		6 (42.9)		5 (35.7)		4 (28.6)		3 (21.4)	
G3/undifferentiated	34	24 (70.6)	0.59	22 (64.7)	0.18	16 (47.1)	0.48	22 (64.7)	0.02	16 (47.1)	0.09
FIGO stages											
I, II	16	9 (56.2)		6 (43.7)		7 (43.8)		4 (25.0)		4 (25.0)	
III, IV	32	23 (71.9)	0.27	22 (68.7)	0.77	14 (43.8)	0.95	22 (68.7)	0.005	15 (46.9)	0.16

When four-fold tables exist, n<40 or T<1; data was analyzed by Fisher’s exact probability test. VEGF, vascular endothelial growth factor; FIGO, International Federation of Gynecology and Obstetrics.

**Table II.** Correlation between the expression of VEGF and its receptors, and hepatic metastasis and ascites in ovarian cancer.

Category	Cases, n	VEGF		Flt-1		KDR		VEGF/Flt-1		VEGF/KDR	
		n (%)	P-value	n (%)	P-value	n (%)	P-value	n (%)	P-value	n (%)	P-value
Lymph node											
(+)	28	24 (85.7)		20 (71.4)		15 (53.4)		20 (71.4)		13 (46.4)	
(−)	20	8 (40.0)	0.009	8 (40.0)	0.02	6 (30.0)	0.1	6 (30.0)	0.005	6 (30.0)	0.25
Hepatic metastasis											
With	8	7 (87.5)		6 (75.0)		7 (87.5)		6 (75.0)		7 (87.5)	
Without	40	25 (62.5)	0.36	22 (55.0)	0.5	14 (35.0)	0.02	20 (50.0)	0.39	12 (30.0)	0.005
Ascites (ml)											
≥1000	30	24 (75.0)		21 (70.0)		16 (53.3)		21		13	
<1000	18	8 (44.4)	0.01	7 (38.9)	0.04	5 (27.8)	0.09	5	0.005	6	0.49

VEGF, vascular endothelial growth factor.

**Table III.** Correlation between the expression of VEGF and its recepteors with MVD in ovarian cancer.

			MVD		
Protein	Expression	Cases, n	Mean	Range	Kruskal-Wallis P-value
VEGF	Positive	32	19.51±8.69	7.2–46.0	
	Negative	16	12.68±4.04	4.1–21.6	0.01
KDR	Positive	21	21.64±8.63	10.2–46.0	
	Negative	27	17.06±7.87	4.1–37.8	0.03
Flt-1	Positive	28	19.19±9.53	4.1–46.0	
	Negative	20	17.16±6.74	8.2–29.4	0.54

VEGF, vascular endothelial growth factor; MVD, microvessel density.
